# Trisomy 9 Mosaicism Diagnosed *In Utero*

**DOI:** 10.1155/2010/379534

**Published:** 2010-07-25

**Authors:** Hironori Takahashi, Satoshi Hayashi, Yumiko Miura, Keiko Tsukamoto, Rika Kosaki, Yushi Itoh, Haruhiko Sago

**Affiliations:** ^1^Department of Maternal-Fetal and Neonatal Medicine, National Center for Child Health and Development, Tokyo 157-8535, Japan; ^2^Department of Clinical Genetics and Molecular Medicine, National Center for Child Health and Development, Tokyo 157-8535, Japan

## Abstract

We present three cases of trisomy 9 mosaicism diagnosed by amniocentesis with ongoing pregnancies after referral to our center due to fetal abnormalities. Two cases were associated with severe fetal growth restriction (FGR), each of which resulted in an intrauterine fetal demise (IUFD) in the third trimester. The other case involved mild FGR with a congenital diaphragmatic hernia and resulted in a live birth with severe development delay. A major prenatal finding of trisomy 9 mosaicism is FGR. Fetuses with trisomy 9 mosaicism can rarely survive in the case of severe FGR.

## 1. Introduction

Trisomy 9 mosaicism is a rare chromosomal abnormality that manifests with multiple anomalies, such as facial, cardiac, osteal, genitourinary, and respiratory abnormalities. More than 50 cases have been reported, most of which were diagnosed after birth. As cases diagnosed prenatally usually culminate in induced abortions [1–4], the natural history of fetuses with trisomy 9 mosaicism remains unknown. We report three cases of trisomy 9 mosaicism diagnosed *in utero* with ongoing pregnancies.

## 2. Case Report

A 36-year-old primigravida was referred to our institute at 29 weeks of gestation because of fetal growth restriction (FGR). A fetal ultrasound examination demonstrated severe asymmetric FGR (<−3.0 standard deviation [SD]) and a single umbilical artery (SUA). An amniocentesis revealed that 27 were normal 46, XX cells and 3 cells (10%) were 47, XX, +9. An intrauterine fetal demise (IUFD) was confirmed at 33 weeks of gestation. The fetus was a 915 g female with a large forehead, a bulbous nose, and micrognathia. The placental weight was 150 g. An autopsy revealed an abnormal lobulation of the right lung.

The second case was that of a 36-year-old primigravida. She was referred to our institute at 31 weeks of gestation due to a left-sided congenital diaphragmatic hernia. The estimated fetal body weight by ultrasound was 1408 g (−1.7 SD). The fetal karyotype by amniocentesis indicated trisomy 9 mosaicism with 29% (6/21 cells) trisomic cells. At 37 weeks of gestation, a 1506 g male was delivered by elective cesarean section. The placenta weighed 350 g. The diaphragmatic hernia was repaired on day 2 of life, followed by a gastrostomy and bronchotomy in the 1st year. Although he had normal G-banding results on postnatal blood karyotyping, interphase FISH performed on abdominal wall muscle tissue obtained during the gastrostomy revealed a mosaic trisomy 9 karyotype. This case has been reported to highlight the cytogenetic discrepancy between amniocytes and postnatal blood [5]. He is now 4 years old and suffers from severe developmental delay.

The third case involved a 41-year-old primigravida. She was referred for evaluation of FGR at 26 weeks of gestation. An ultrasound examination revealed severe asymmetric FGR 530 g (−3.2 SD) and an SUA. Chromosomes from amniocentesis revealed a mosaic trisomy 9 constitution, as follows: 46, XX (18 cells)/47, XX, +9 (11 cells). An IUFD was confirmed at 38 weeks of gestation. The fetus was a 1220 g female. The placenta weighed 107 g. An autopsy revealed no major abnormalities except facial dysmorphism with a bulbous nose, micrognathia, and low-set ears.

## 3. Discussion

We have reported three cases of trisomy 9 mosaicism diagnosed by amniocentesis with ongoing pregnancies. FGR was observed prenatally, and specific facial findings were observed after birth in all the cases. Two of three cases, which showed severe FGR and SUA, resulted in IUFDs in the third trimester. The other case with mild FGR resulted in a live birth and had severe developmental delay. Fetuses with trisomy 9 mosaicism in which the major prenatal finding is FGR rarely survive, even though they have no major anomalies.

Trisomy 9 mosaicism manifests with various abnormalities. Facial anomalies, such as low-set ears, micrognathia, and bulbous nose, are universal. Cleft lip is expressed in about 20% of cases [6]. Orthopaedic abnormalities, including hip dislocations and arthrogryposis, are also highly expressed. Wide-ranging malformations, such as congenital heart disease, intracerebral lesions, and urogenital abnormalities, are also observed (Table 1). Our three cases had common specific facial findings, such as micrognathia and bulbous noses. Other malformations included a congenital diaphragmatic hernia and an abnormal lobulation of the lung; these visceral abnormalities are rare.

Postpartum cytogenetic conformation of trisomy 9 mosaicism was performed in only one case. If mosaicism is detected in any one tissue, it is expected that different tissues will have different levels of mosaicism and consequently the clinical presentation of each case may vary considerably. The case which underwent cytogenetic analysis postnatally revealed cytogenetic discrepancies between the tissues [5]. There was a normal karyotype in the blood and trisomy 9 mosaicism in the abdominal wall tissue. In this case, the infant demonstrated mild FGR with a congenital diaphragmatic hernia. When a cytogenetic discrepancy between amniocytes and postnatal blood is observed, it is not rare for mosaic tissue to be confined to a specific organ [5, 7]. We failed to perform postnatal cytogenetic analysis on the other two cases which resulted in IUFDs. It is difficult to obtain usual G-banding karyotype results on specimens from stillborn infants. However, these two cases were coincident with a phenotype of trisomy 9 mosaicism, such as a specific facial appearance, and the two stillborn infants were considered to have trisomy 9 mosaicism. 

Our search revealed 20 reported prenatal cases of trisomy 9 mosaicism [3, 8–12]. Of the 20 cases, 11 resulted in induced abortions, and the pregnancies were not interrupted in the remaining 9 cases (Table 2). Of these 9 cases, 8 led to live births and 1 case resulted in an IUFD [12]. In contrast, two of three cases resulted in IUFDs in this report. In general, many fetuses with common trisomies are lost before birth. In terms of trisomy 21, which is the most common aneuploidy, the estimated fetal death rate is 30% between the 2nd trimester and term [13]. Chromosomal abnormalities were present in 38% of anomalous stillborns, and 4.6% of morphologically normal fetuses of 750 stillbirths [14]. It is not surprising, therefore, that two of three cases of trisomy 9 mosaicism resulted in IUFDs. The main feature of trisomy 9 mosaicism IUFDs is severe growth restriction. Two of the three cases we evaluated had severe FGR without major congenital abnormalities and resulted in IUFDs. According to reported 12 cases including our own cases (Table 2), three cases that resulted in IUFDs involved severe FGR. Adversely, the other 9 cases of liveborn did not reveal severe FGR. Arnold reviewed 23 cases of trisomy 9 mosaicism; most were diagnosed postnatally with an average birth weight of 2690 g, which is consistent with mild FGR [6]. In addition, reported cases incidentally detected by midtrimester routine amniocentesis associated with maternal advanced age led to live births uneventfully (Table 2). Trisomy 9 mosaicism can result in IUFDs when there is severe FGR, even though there are no structural abnormalities. However, it is still unknown whether the degree of FGR correlates with IUFDs in chromosomal abnormalities. 

Our cases contribute to clarifying the natural history of trisomy 9 mosaicism diagnosed *in utero*. Trisomy 9 mosaicism fetuses with severe FGR can rarely survive. These findings are helpful for genetic counseling for trisomy 9 mosaicism diagnosed.

##  Conflict of Interest

The authors declare no conflicts of interest.

## Figures and Tables

**Table 1:** Clinical findings of trisomy 9 mosaicism with high frequency.

*Prenatally*
Fetal growth restriction
Ventricular septal defect
Micrognathia
Single umbilical artery
Amniotic fluid disorders

*Postnatally*
Craniofacial abnormalities
Wide fontanels
Microphthalmia
Bulbous nose
Low-set ears
Micrognathia
Heart abnormalities
Ventricular septal defect
Patent ductus arteriosus
Genitourinary malformations
Hydronephrosis
Microkidneys
Musculoskeletal abnormalities
Dislocated hips
Joint limitation
Overlapping fingers
Abnormal ossification

**Table 2:** Characteristics and outcomes of reported cases of prenatal trisomy 9 mosaicism.

Case	Reference	Maternal age (years)	Time of exam (weeks)	Trigger of detection	FGR	Other fetal findings	Outcomes
1	Bureau et al. [8]	24	36	CNS anomaly	−	Dandy-Walker variant	Liveborn, death at 2 weeks
2	Saura et al. [3]	28	30	PDA, polyhydramnios	−	PDA	Liveborn at 35 weeks
3		29	33	FGR	+	—	Alive at 21 months; severe developmental delay
4	Greenberg et al. [9]	39	16	AAMA	−	—	Liveborn at term
5	Hsu et al. [10]	48	NA	AAMA	−	—	Normal development at 3 years, 8 months
6		42	NA	AAMA	−	—	Liveborn at 23 weeks; death at 4 days
7		38	NA	AAMA	+	Multiple anomalies (no details)	Liveborn at term
8	Sherer et al. [11]	20	31	FGR	Mild	Right hydronephrosis	liveborn, death at 6 weeks
9	Smoleniec et al. [12]	28	34	Severe FGR	Severe	—	IUFD at 34 weeks
10	Case 1	35	29	Severe FGR	Severe	—	IUFD at 33 weeks
11	Case 2 in [5]	36	31	CDH	Mild	Left-sided congenital diaphragmatic hernia	Alive at 4 years; severe developmental delay
12	Case 3	41	24	Severe FGR	Severe	—	IUFD at 38 weeks

AAMA: amniocentesis with advanced maternal age; CNS: central nerve system; FGR: fetal growth restriction; IUFD: intrauterine fetal demise; PDA: patent ductus arteriosus; NA: not available.
